# Clonal array profiling of scFv-displaying phages for high-throughput discovery of affinity-matured antibody mutants

**DOI:** 10.1038/s41598-020-71037-3

**Published:** 2020-08-24

**Authors:** Yuki Kiguchi, Hiroyuki Oyama, Izumi Morita, Mai Morikawa, Asuka Nakano, Wakana Fujihara, Yukari Inoue, Megumi Sasaki, Yuki Saijo, Yuki Kanemoto, Kaho Murayama, Yuki Baba, Atsuko Takeuchi, Norihiro Kobayashi

**Affiliations:** Kobe Pharmaceutical University, 4-19-1, Motoyama-Kitamachi, Higashinada-ku, Kobe, 658-8558 Japan

**Keywords:** Biotechnology, Immunology, Molecular biology

## Abstract

"Antibody-breeding" approach potentially generates therapeutic/diagnostic antibody mutants with greater performance than native antibodies. Therein, antibody fragments (e.g., single-chain Fv fragments; scFvs) with a variety of mutations are displayed on bacteriophage to generate diverse phage-antibody libraries. Rare clones with improved functions are then selected via panning against immobilized or tagged target antigens. However, this selection process often ended unsuccessful, mainly due to the biased propagation of phage-antibody clones and the competition with a large excess of undesirable clones with weaker affinities. To break radically from such panning-inherent problems, we developed a novel method, clonal array profiling of scFv-displaying phages (CAP), in which colonies of the initial bacterial libraries are examined one-by-one in microwells. Progenies of scFv-displaying phages generated are, if show sufficient affinity to target antigen, captured in the microwell via pre-coated antigen and detected using a luciferase-fused anti-phage scFv. The advantage of CAP was evidenced by its application with a small error-prone-PCR-based library (~ 10^5^ colonies) of anti-cortisol scFvs. Only two operations, each surveying only ~ 3% of the library (9,400 colonies), provided five mutants showing 32–63-fold improved *K*_a_ values (> 10^10^ M^−1^), compared with the wild-type scFv (*K*_a_ = 3.8 × 10^8^ M^−1^), none of which could be recovered via conventional panning procedures operated for the entire library.

## Introduction

**Figure 1:**
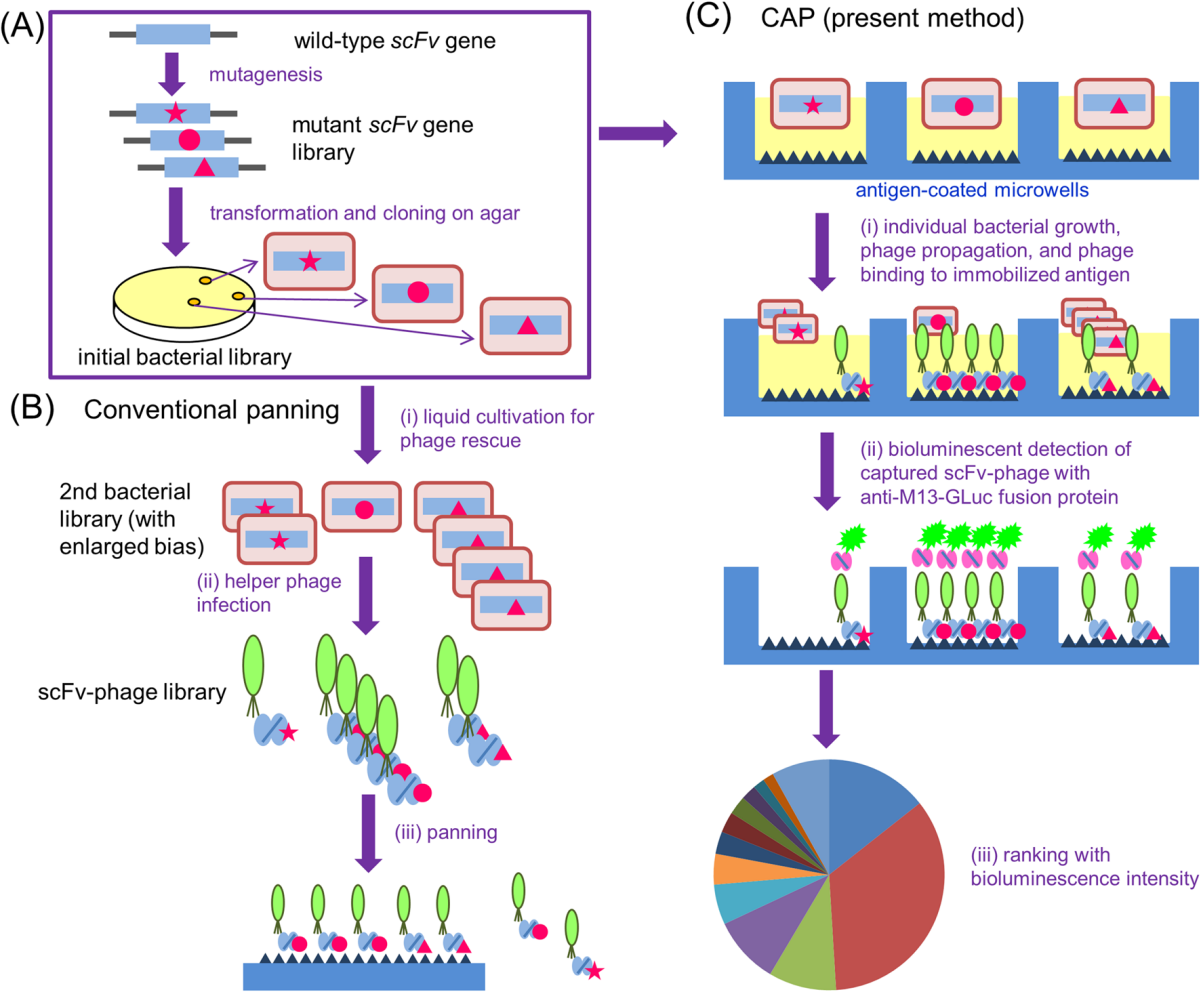
Principle and advantages of CAP compared with conventional panning. **(A)** As common steps between both the methods, *E. coli* cells are transformed with mutagenized *scFv* genes and grown overnight on agar to serve as the initial transformant library with “undisturbed” diversity. **(B)** Conventional panning starts from re-cultivation of the bacterial library in liquid medium for phage rescue, which increases clonal bias, mainly due to differences in their propagation abilities. The resulting “polyclonal” scFv-phages react competitively with a limited number of immobilized antigens. **(C)** In CAP, the bacterial clones are individually cultivated in microwells containing the helper phage. Therein, each clone propagates and produces scFv-phages concurrently without competing with different clones. The scFv-phages specific to the aimed antigen bind the pre-immobilized antigen and are detected in a bioluminescence assay. The signal-intensity data obtained from the microwells are ranked and captured phages in selected wells with high intensity are recovered and propagated for subsequent analysis for antigen-binding abilities.

As shown by the feat that the pioneers in this field received Novel Prize in Chemistry in 2018^1^, the in vitro molecular evolution revolutionalized in generating therapeutic and diagnostic antibodies with artificial sequences that might show advanced characteristics compared with native antibodies (“antibody-breeding”)^2–5^. Typically, smaller fragments (e.g., single-chain Fv fragments; scFvs) of prototype antibodies are mutagenized to generate a diverse library of mutants (Fig. 1A). Then, genotype–phenotype-connecting systems play a vital role in isolating rarely appearing evolved species. Among the systems developed, the phage display used the most because of its easier handling and availability of the necessary materials^6–8^. Therein, mutant scFvs are expressed on Ff bacteriophage (e.g., M13 phage) particles, as fusion proteins usually with the minor coat protein pIII (Fig. 1B). The scFv-displaying phages (scFv-phages) with improved antigen-binding characteristics are commonly isolated via panning, wherein all the library members are simultaneously reacted with a limited amount of immobilized or tagged antigens (Fig. 1B). The goal is to recover desirable species as the bound fraction, which are then proliferated by infecting bacteria for subsequent panning or characterization.

**Figure 2:**
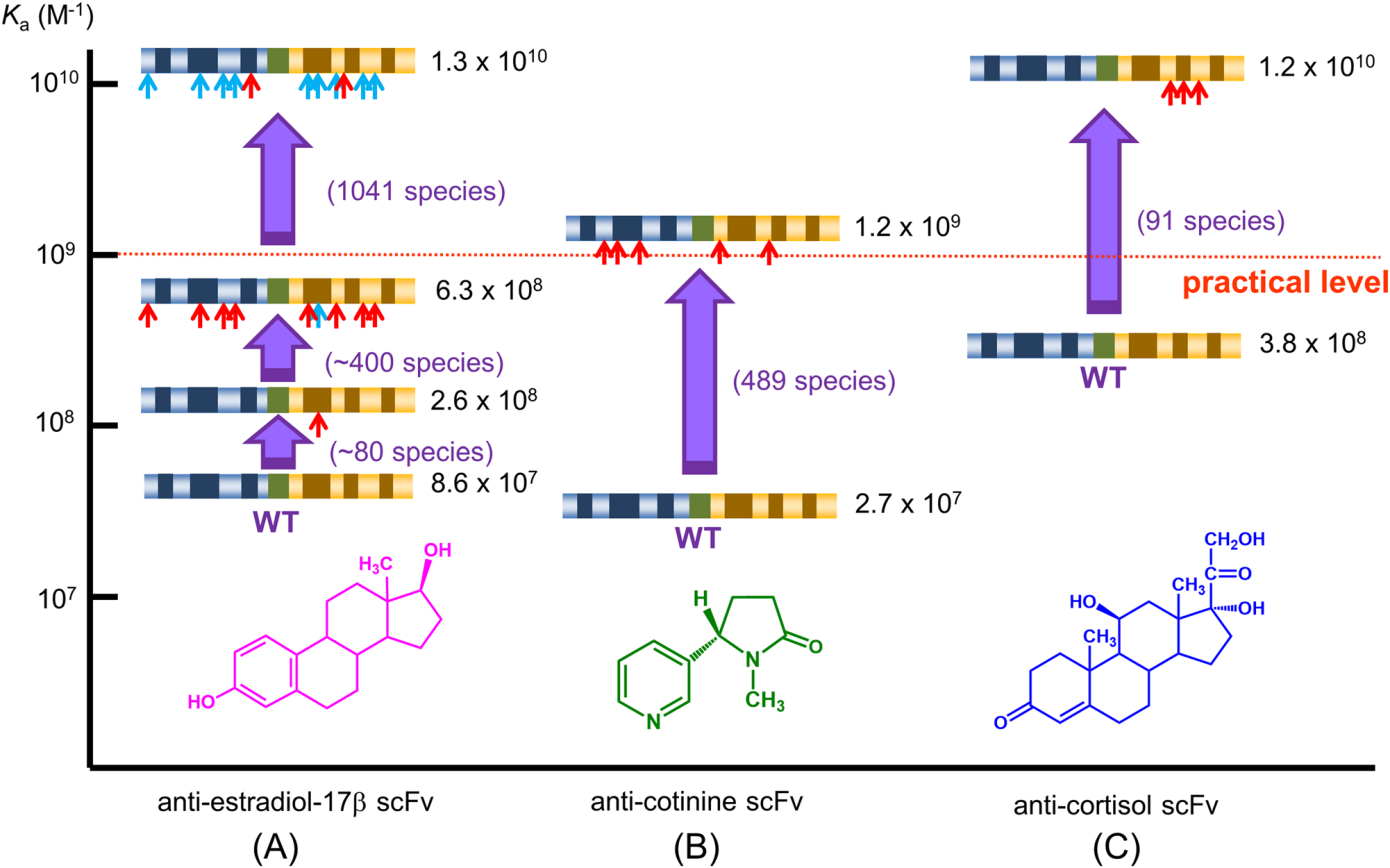
Summary of our previous affinity-maturation experiments for scFvs based on phage display. The process for isolating affinity-matured scFvs against **(A)** estradiol-17β^12–14^, **(B)** cotinine^15^, and **(C)** cortisol^16^ is schematically illustrated with the primary scFv structures, in which the red arrows represent newly-introduced amino acid substitutions and the blue arrows represent already-introduced substitutions. *K*_a_ values are shown on the ordinate. The numbers of clones examined until we found the most improved scFv are shown in parentheses. In these experiments, the improved scFv-phages were selected by panning using the polystyrene immunotubes (12 × 75 mm; Nunc MaxiSorp). The binding and elution methods used therein are summarized as follows. *For anti-estradiol-17β (E*_*2*_*) scFv*: 1st mutagenesis, 5 rounds using immunotubes coated with E_2_-BSA (10 µg/mL), washed 3, 3, 10, 20, and 20 times, and bound phages were eluted with 0.1 M triethylamine (TEA); 2nd mutagenesis, 3 rounds using immunotubes coated with E_2_-BSA (1 µg/mL), washed 20 times each, and eluted with TAE, TAE, and E_2_ (10 ~ 0.01 mol eq to immobilized E_2_); 3rd mutagenesis; 3 rounds using E_2_-cleavable biotin conjugate (40 ng/mL)/immunotubes coated with NeutrAvidin (50 µg/mL), washed 20 times each, and eluted with dithiothreitol. *For anti-cotinine scFv:* 3 rounds using immunotubes coated with cotinine-BSA (100 µg/mL), washed 3, 3, and 10 times, and bound phages were eluted with cotinine (10, 10, 1 mol eq to immobilized cotinine). *For anti-cortisol scFv:* 3 rounds using immunotubes coated with CS-BSA (0.5 µg/mL), washed 3, 3, and 5 times, and bound phages were eluted with cortisol (10, 1, and 0.1 ng).

However, the panning-based selection process often fails to straightforward discovery of improved scFv-phages even after extensive efforts. As the reason for this crucial problem, we should not disregard for biasness for propagating transformants as well as for infection/replication of phage clones^9^ and competition with a large excess of undesirable mutants with weaker affinities against target antigens (Fig. 1B). To enable more sensitive immunochemical analyses^10,11^, we have generated affinity-matured scFv mutants against estradiol-17β^12–14^, cotinine^15^, and cortisol^16^, each of which gained an equilibrium affinity constant (*K*_a_) enhanced by > 150-fold, > 40-fold, and > 30-fold, respectively (Fig. 2A–C). These improved binders were all isolated by the panning, but with different protocols arranged after trial and error. However, we had to examine the affinity of > 1,000, ~ 500, and ~ 50 scFv-phage clones in detail until we discovered the desirable anti-estradiol-17β, anti-cotinine, and anti-cortisol scFv mutant, respectively, despite that the tested clones were the members of “enriched” populations. It should be noted that acceptably efficient selection was achieved only for isolation of the anti-cortisol scFv (Fig. 2C)^16^ that was performed with the simplest panning protocol among the three instances.

Such panning-inherent limitations should be overcome by screening individual phage clones generated from the initial bacterial libraries that maintain the original diversity, and not from panning-enriched libraries. Thus, we devised “clonal array profiling of scFv-displaying phages (CAP),” a reliable and robust system for recovering rare affinity-matured scFv phages that does not require sophisticated machinery or intelligent technology. This system, schematically illustrated in Fig. 1C, directly (i.e., without any enrichment step) provided a set of scFv mutants showing 32–63-fold improved and 10^10^-order *K*_a_ values against a small biomarker cortisol (hydrocortisone; Fig. 2C and Supplementary Fig. S1), contained in a small (~ 10^5^-order) scFv library generated via a simple mutagenesis based on error-prone polymerase chain reaction (PCR). None of such improved species could be recovered via a multiple operation of panning including three different protocols. We then applied CAP to a related but different scFv library, and again succeeded in isolating affinity-matured mutants with a minimum experimental effort.

## Results

### Basic principle of CAP

In our CAP, we examined the colonies of the initial bacterial library grown on agar plates one-by-one in different microwells, so as not to disturb their original diversity or compete with the different clones (Fig. 1C). TG1 strain of *Escherichia coli* (*E. coli*) was transformed with a mutant *scFv* gene library and spread on agar plates. The colonies grown on agar were transferred individually into microwells in 96-well plates pre-coated with the target antigen and filled with liquid medium containing the KM13 helper phage^17^. The plates were then incubated, and antigen-specific scFv-phages rescued therein were, if present with enough affinity and/or copy numbers, captured on the microwells and detected with the bioluminescent assay using an in-house-prepared fusion protein linking an scFv against M13 phage (specific to the pVIII major coat protein)^18^ and *Gaussia* luciferase^19^ (anti-M13-GLuc) (Supplementary Fig. S2A–C). We confirmed that the virions with 1.8 × 10^10^ plaque forming units (pfu) were generated in a single microwell (according to the average of tested 58 colonies), and could be easily detected using anti-M13-GLuc (Supplementary Fig. S2C). For more selective isolation of the higher affinity mutants, a variation of CAP containing “off-rate-dependent (ORD) selection” process was performed in parallel with the standard CAP procedure, which is described later.

### Comparison of CAP and the conventional panning

**Figure 3:**
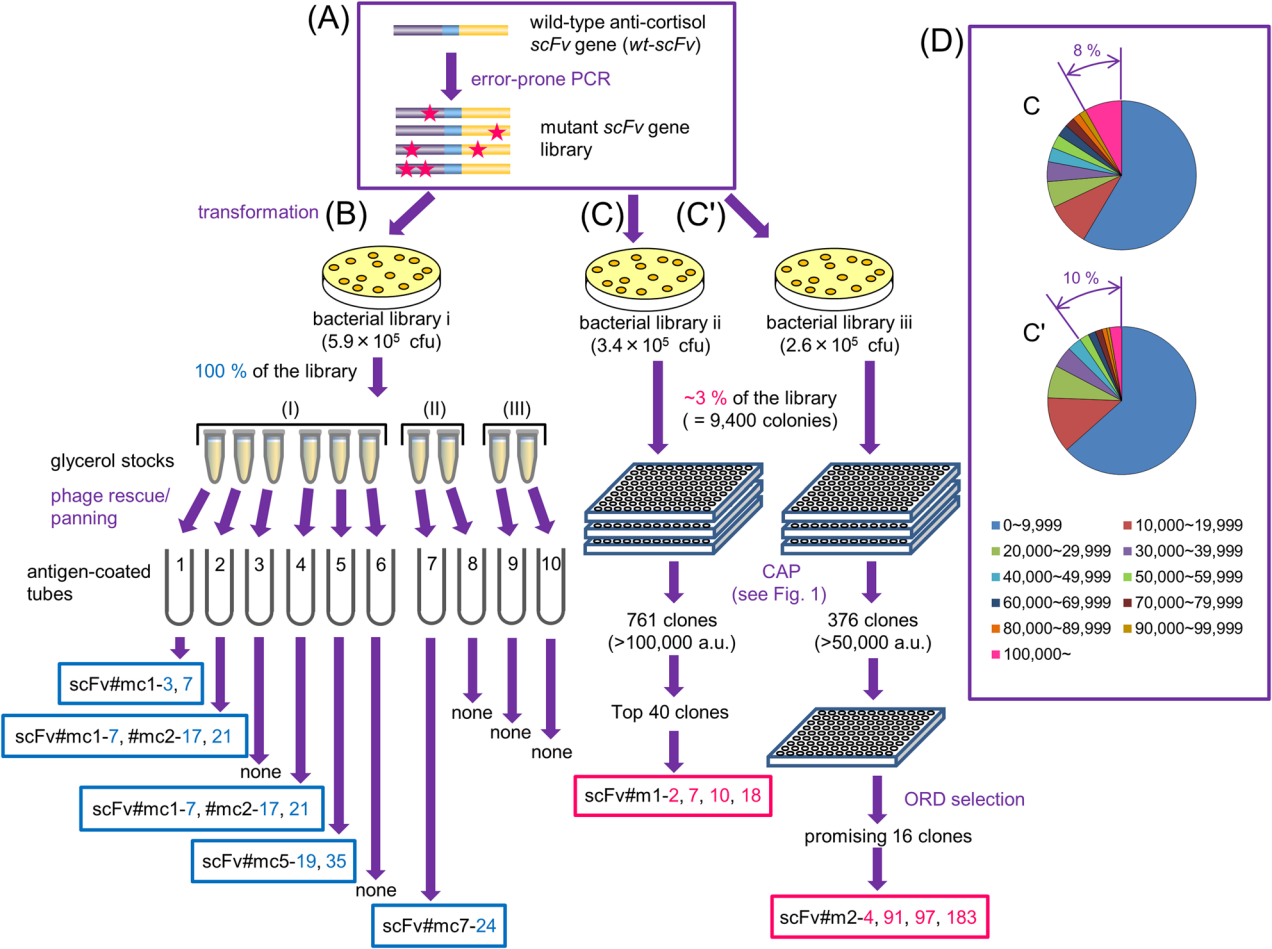
Workflow for comparative studies between the present CAP and conventional panning. **(A)** The same anti-cortisol *scFv* library, ligated in the phagemid vector, was used to generate bacterial libraries i–iii with similar transformant numbers (~ 10^5^ colonies). **(B)** Conventional panning was performed as a parallel ten experiment against the phages generated from all the transformants in the library i according to the protocol (I), (II), or (III) as shown in the text. **(C, C')** Approximately 3% of all the colonies from libraries ii and iii was subjected to CAP without **(C)** or with (**C')** off-rate-dependent (ORD) selection. **(D)** The appearance frequency of bioluminescence intensity [arbitrary units (a.u.)] values for the 9,400 tested clones, observed in CAP without (**C**, upper) or with (**C'**, lower) ORD selection.

A comparative study was performed according to the workflow shown in Fig. 3. As a target for the testing, a small and simple gene library (randomly-mutated library) was generated by a single random mutagenesis with error-prone PCR^12–16,20^ throughout the gene fragment encoding the wild-type scFv against cortisol (wt-scFv), which was composed of the non-mutated heavy and light chain variable domains (V_H_ and V_L_) of a mouse anti-cortisol antibody (Ab#3)^16^ (Fig. 3A).

### a. Panning

TG1 cells were transformed with these mutant *scFv* genes, and the resulting bacterial library that initially contained 5.9 × 10^5^ transformants as colony-forming unit (cfu) was cultivated 1 h and equally divided to make multiple glycerol stocks. These were separately used for a standard phage rescue with the KM13 helper phage, and the generated virions were submitted to one of three kinds of panning (I–III), each of which was performed with three rounds using polystyrene tubes (immunotubes) coated with a conjugate of cortisol and bovine serum albumin (CS-BSA)^19^ (Fig. 3B) as follows. (I) Binding reaction of phages was performed against the immunotubes immobilized with a constant concentration of CS-BSA (#1–6). After washing with a constant stringency, bound phages were eluted with a standard acidic (pH 2.2; #1–3) or basic (pH 12; #4–6) condition. (II) Binding reaction was performed against the immunotubes immobilized with gradually reduced concentrations of CS-BSA (#7, 8). After washing with a gradually increased stringency, bound phages were eluted with the acidic (pH 2.2) condition. (III) Binding reaction was performed using the immunotubes immobilized with a constant concentration of CS-BSA (#9, 10). After washing with a constant stringency, bound phages were eluted by addition of cortisol solutions with gradually reduced concentrations: this protocol previously succeeded in isolating the improved anti-cortisol scFv as referred to above^16^ (Fig. 2C).

**Figure 4:**
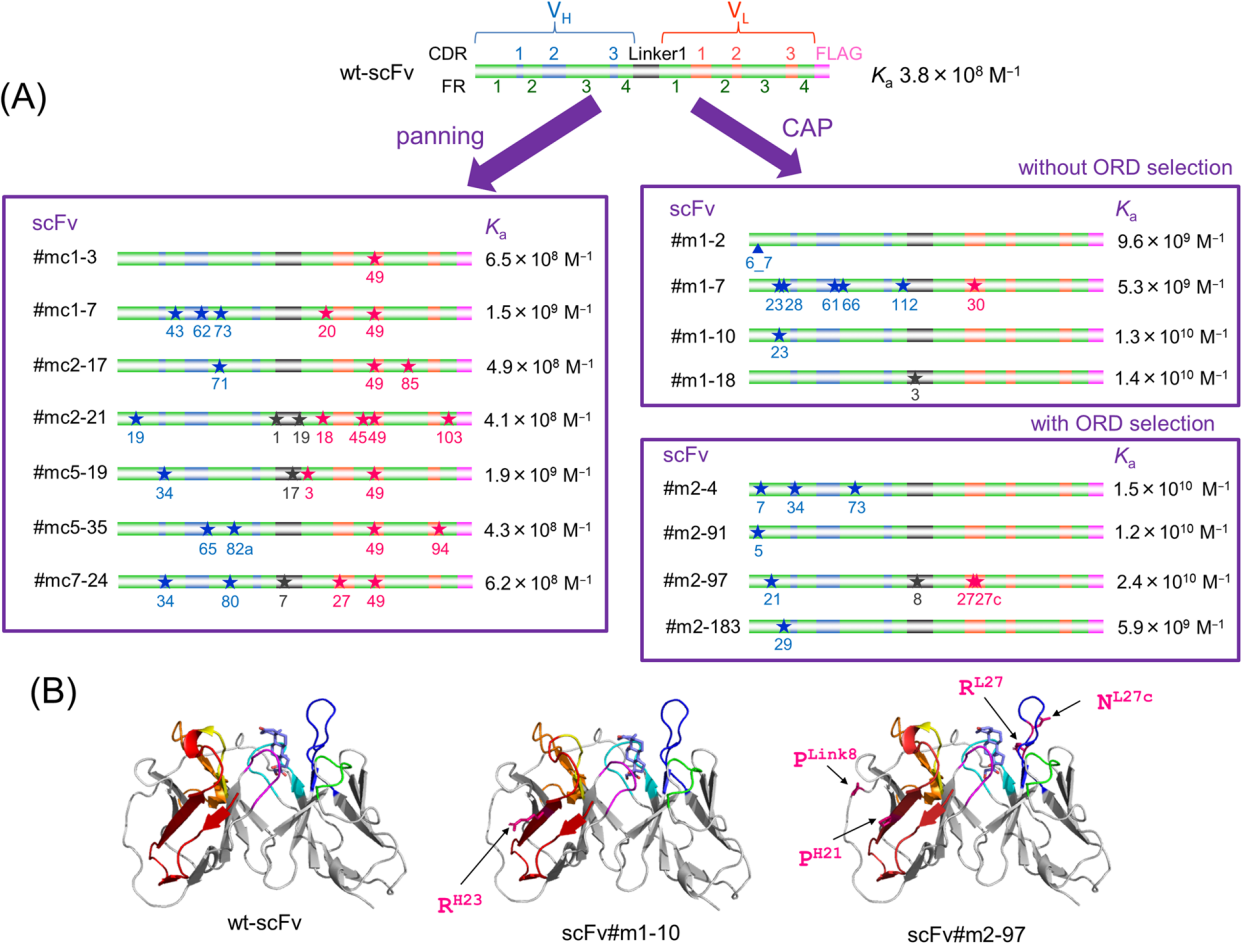
Structures and affinities of the improved scFvs. **(A)** A schematic illustration of the primary structures and *K*_a_ values of the improved scFvs obtained via conventional panning (left box) and CAP method (right boxes) without (upper box) or with (lower box) ORD selection. Amino acid sequences were deduced from the nucleotide sequences and the numbering for the V_H_ and V_L_ domains was based on the Kabat definition^22^. In wt-scFv, the V_H_ and V_L_ domains (sequences of which were referred to in our previous report)^16^ were combined via a “linker1” sequence composed of the following 19 amino acids: VSS(GGGGS)_3_ T. The stars and arrowhead denote substitutions and insertion, respectively. Details are as follows: (left box) scFv#mc1-3 (V_L_) C49G; #mc1-7 (V_H_) Q43R, K62*(amber codon; readthrough as Q), K73R, (V_L_) T20A, C49S; #mc2-17 (V_H_) V71I, (V_L_) C49S, T85A; #mc2-21 (V_H_) K19R, (linker) V1G, T19A, (V_L_) R18G, K45R, C49S, K103*(amber codon; readthrough as Q); #mc5-19 (V_H_) M34T, (linker) G17V, (V_L_) V3A, C49G; #mc5-35 (V_H_) D65G, I82aN, (V_L_) C49Y, L94P; #mc7-24 (V_H_) M34V, M80L, (linker) G7C, (V_L_) K27R, C49S; (right upper box) scFv#m1-2 (V_H_) Q6-P7*(insertion of amber codon between Q6 and P7; readthrough as Q); #m1-7 (V_H_) K23R, T28S, Q61P, K66R, S112F, (V_L_) Y30H; #m1-10 (V_H_) K23R, W36*(opal codon; readthrough as W); #m1-18 (linker) S3G; (right lower box) scFv#m2-4 (V_H_) P7S, M34T, K73E; #m2-91 (V_H_) Q5L; #m2-97 (V_H_) S21P, (linker) S8P, (V_L_) K27R, S27cN; #m2-183 (V_H_) F29L. **(B)** Protein ribbon structures for wt-scFv, scFv#m1-10, and #m2-97 were constructed using the SWISS-MODEL Protein Modelling Server^46^, and their conformations when docked to cortisol were predicted using SwissDock^47^. In the ribbon representation of the scFv backbones, V_H_-CDR1 (yellow), V_H_-CDR2 (orange), V_H_-CDR3 (magenta), V_L_-CDR1 (dark blue), V_L_-CDR2 (light green), and V_L_-CDR3 (light blue) are shown with β-sheet structures (bold gray arrows), in which FR1 is denoted with red shading. The substituted amino acids are also indicated in red.

Among the recovered scFv-phages from each tube, 50 clones (thus, total 500 clones) were analyzed with the phage-targeted enzyme-linked immunosorbent assay (ELISA)^12–16^. The 34 positive clones that showed bound absorbance of > 0.3 were converted to the soluble (non-phage-linked) scFv form, and their *K*_a_ values against free (not immobilized) cortisol were determined by the Scatchard analysis^12–16,21^. Only seven mutants had improved *K*_a_ compared with wt-scFv (*K*_a_, 3.8 × 10^8^ M^−1^), however, the greatest *K*_a_ of which (1.9 × 10^9^ M^−1^) was only 5.0-fold higher than that of wt-scFv (Fig. 4A). Unexpectedly, the highest (aforementioned) and second-highest *K*_a_ (1.5 × 10^9^ M^−1^) were recorded with the mutants (scFv#mc5-19 and 1-7) both isolated from the panning (I) performed with the mildest stringency. The panning (II) with a standard protocol with the gradually-increased stringency provided only a poorly improved binder (scFv#mc7-24) showing a *K*_a_ in the 10^8^ M^−1^ range. The panning (III), previously succeeded in isolating the affinity-matured anti-cortisol scFv from a similar library, generated no improved mutant.

The panning (I), performed simultaneously in sextuplicate, revealed difficulty in obtaining reproducible results: i.e., the clones scFv#mc1-7, 2-17, and 2-21 appeared from two or more different panning tubes, while the tubes #3 and #6 showed no improved clones (Fig. 3B). For these seven mutants, the number of substitutions per entire scFv molecule ranged from 1 to 7 positions with the average of 4.1. Proportions of these substitutions occurred in the V_H_ and in the complementarity-determining regions (CDRs) were 40% and 24%, respectively (calculated excluding those in the linker).

The most remarkable feature observed for the panning-derived mutants was the fact that each of the seven mutants contained an amino acid substitution at the V_L_49 position that eliminated the original cysteine (C), which was rather unusual because C residues are rarely observed in the antibody variable regions, except for those highly conserved at the V_H_22 and V_H_92, V_L_23 and V_L_88, each pair of which forms the intra-chain disulfide bonds^22^.

### b. CAP

Using the same *scFv* gene library as above, a bacterial library with a similar size (3.4 × 10^5^ transformants) was obtained, from which 9,400 transformants (corresponding to ~ 3%) were directly submitted to CAP using 100 microplates with manual handling (Fig. 3C). From the 761 clones that generated > 100,000 arbitrary units (a.u.) of bioluminescence (~ 8% of 9,400 clones; Fig. 3D), the top 40 clones were selected and the relevant virions were retrieved from the microwells to examine the binding ability in the phage-targeted ELISA (Fig. 3C). Seven species (scFv#m1-1, 2, 7, 8, 10, 18, and 19) that showed stronger inhibition after adding the cortisol standards were converted to soluble scFvs. Four out of the seven scFvs (scFv#m1-2, 7, 10, and 18) showed 14–37-fold enhanced *K*_a_ (0.53–1.4 × 10^10^ M^−1^) versus wt-scFv [Fig. 4A (right/upper rectangle); Scatchard plots^21^ are in Supplementary Fig. S3A]. We should emphasize here the excellent efficiency that enabled generation of the four improved mutants from the top 40 clones. Because these top 40 were selected from ~ 3% of the library, we could estimate the remaining ~ 97% might contain an additional 4 × (97/3) affinity-matured species.

To increase the efficiency of obtaining affinity-matured phage-scFvs, ORD selection, which might preferentially isolate the mutants with slower dissociation (i.e., lower off-rate)^23^, was employed in this profiling system (Fig. 3C'). The 376 scFv-phages that showed strong luminescence (~ 10% of the tested 9,400 clones) (Fig. 3D) were propagated in different plates to allow for their “initial binding” to the immobilized cortisol. After removing unbound materials, the microwells were incubated with a solution of 14 µM free cortisol for 4 h, and then the supernatant (potentially containing scFv-phages that had been dissociated and complexed with free cortisol) was removed again. In these incubation steps, we set the molar ratio of the free cortisol added versus the immobilized cortisol-residues to be 300:1 to prevent re-binding of dissociated virions to the immobilized cortisol residues. The incubation period employed here was near the *t*_1/2_ of scFvs with the *k*_d_ of *ca*. 5 × 10^−5^ s^−1^. This cycle was repeated three times and the bioluminescence was measured after each removal. Sixteen scFv-phage clones that still showed significant luminescence (Supplementary Fig. S4) were examined for their cortisol-binding ability. Four out of the 16 clones were found to display scFvs (scFv#m2-4, 91, 97, and 183; thus, higher probability than the without ORD selection) that showed 16–63-fold higher *K*_a_ (0.59–2.4 × 10^10^ M^−1^) than wt-scFv, as the soluble form [Fig. 4A (right/lower rectangle); Scatchard plots are in Supplementary Fig. S3B]. Indeed, bio-layer interferometry (BLI) analysis^24^ showed that these mutants had lower dissociation rate constants (*k*_d_) than that of wt-scFv (2.4 × 10^−3^ s^−1^): i.e., for scFv#m2-4, 91, 97, and 183, the *k*_d_ values were 0.38, 0.21, 0.87, and 2.0 × 10^−3^ s^−1^, respectively (average of 0.87 × 10^−3^ s^−1^).

The primary structures indicated that the improved mutants obtained here were quite different from those obtained with the panning (Fig. 4A). Substitutions and insertions were lesser (average number was 2.3), the proportions of which in the V_H_ (81%) and in the CDRs (31%) (calculated as described above) were obviously higher than those found in the panning-derived mutants. One of the most notable aspects, however, was the fact that the five mutants gained 16–37-fold greater *K*_a_ as the result of only a single substitution (scFv#m1-10, 18, #m2-91, and 183) or a single insertion (scFv#m1-2). Furthermore, four of the five scFvs (scFv#m1-2, 10, scFv#m2-91, and 183) were modified at their V_H_ framework region (FR) 1. Three mutants with multiple substitutions (scFv#m1-7, #m2-4 and 97) also substituted at the V_H_-FR1. The protein modelling docked with cortisol was shown for the FR1-modified mutants (scFv#m1-10 and #m2-97) with wt-scFv, in which the FR1 sequences were highlighted (Fig. 4B). However, prominent alterations in the backbone and CDR conformations were not observed after the substitutions. We should pay attention to one more mutant scFv#m1-18. This gained 37-fold greater *K*_a_ as a result of a single serine(S) → glycine(G) substitution at the third residue of the linker combining the V_H_ and V_L_ domains: the original sequence of which was the following 19 residues as V(valine)SS(GGGGS)_3_ T(threonine), and the underlined S was substituted.

It was also noteworthy that scFv#m1-2 and 10 referred above were the products of *scFv* genes containing a nonsense codon. Thus, the gene encoding scFv#m1-2 had an insertion of amber (TAG) codon between the codons for the V_H_6 and V_H_7 amino acids, while the gene encoding scFv#1-10 had an opal (TGA) codon introduced by a G → A transition altering the original TGG codon that encoded the V_H_36 tryptophan (W). Such a nonsense mutation was also found in the gene encoding the panning-derived mutants (scFv#mc1-7 and 2-21; Fig. 4A). Since these scFvs bound to cortisol with significant *K*_a_ values (over 10^8^ M^−1^), the nonsense codons should be readthrough to generate full-length scFv molecules. We identified the incorporated amino acids by Edman sequencing for scFv#m1-2 and liquid chromatography-tandem mass spectrometry (LC/MS/MS) fingerprinting for scFv#m1-10, mc1-7, and 2-21 (Supplementary Fig. S5), both performed on affinity-purified soluble-form scFv proteins: the amber codons in scFv#m1-2, mc1-7, and 2-21 were decoded as glutamine (Q), and opal codon in scFv#m1-10 was decoded as W, respectively, as expected from the previously shown stop codon readthrough in bacteria (Nonsense suppressor-EcoliWiki, https://ecoliwiki.org/colipedia/index.php/Nonsense_suppressor). Consequently, the V_H_36 amino acid in scFv#m1-10 was found not to be substituted, maintaining the original residue in wt-scFv (i.e., W).

### Antigen-binding performance of affinity-matured scFv mutants

**Figure 5:**
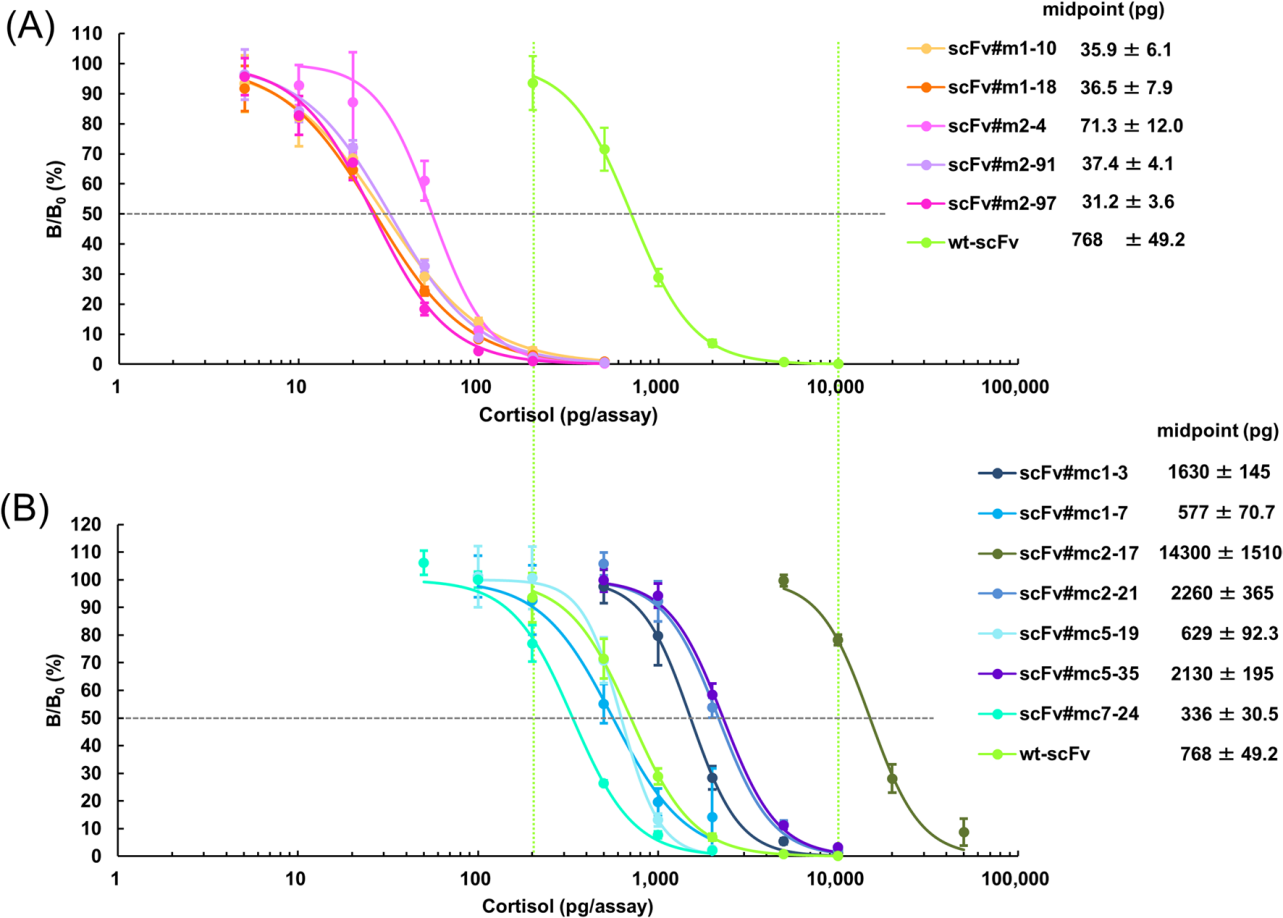
Typical dose–response curves for cortisol in competitive ELISAs using wt-scFv and the improved scFvs obtained with **(A)** CAP or **(B)** the conventional panning. The vertical bars indicate the SD for intra-assay variance (*n* = 4). The midpoint values (mean ± SD for inter-assay variance; *n* = 4) are listed together. In these assays, the scFv concentrations were adjusted to give bound enzyme activities at B_0_ (the reaction without cortisol standard) of approximately 1.0–1.5 absorbance after a 30-min enzyme reaction. The background absorbance (observed without addition of scFvs) was lower than 5.0% of the B_0_ absorbance.

Cortisol is used as a biomarker for the functions of the hypothalamic–pituitary–adrenal axis^25^ and thus practical anti-cortisol antibodies have always been in great demand as diagnostic reagents. However, only a few publications have demonstrated the production of monoclonal antibodies capable of targeting serum or urinary cortisol^26–28^. Immunoassay sensitivities closely correlate with affinities of the antibodies used^10^. As expected, the five mutants with a *K*_a_ of > 10^10^ M^−1^ isolated from the randomly-mutated library with CAP (scFv#m1-10, 18, #m2-4, 91, and 97) showed dramatically enhanced sensitivities in competitive ELISAs, as shown by 11–25-fold lower midpoint values (31–71 pg/assay) than those of wt-scFv (768 pg/assay) in dose–response curves (Fig. 5A). Among the panning-derived mutants, scFv#mc7-24 isolated from the protocol (II) with the fourth highest *K*_a_ among the seven mutants showed the most increased sensitivities with 2.3-fold lower midpoint values, but was not as sensitive as the CAP-derived mutants (Fig. 4B). The six mutants from the protocol (I) exhibited only slightly increased (scFv#mc1-7 and #mc5-9; ~ 1.3 and 1.2-fold as midpoint values) or a rather deteriorated response (the other four scFvs) despite their obviously improved *K*_a_ values (Fig. 4A). We have no clear explanation for these contradicting results now, but this might be attributable to a possible feature of these mutants to recognize the bridge (linker) structure connecting cortisol and BSA in the conjugate coated on the microplates^29^.

The detection limit of the ELISAs using the four mutants that exhibited midpoints of less than 50 pg/assay, i.e., scFv#m1-10, 18, #m2-91, and 97, was 5.5, 11, 8.1, and 9.0 pg/assay, respectively, based on the definition as the cortisol amount that provided a bound signals two standard deviations (SDs) below the average (*n* = 10) of the signals at zero concentration; which corresponds to 1.1–2.2 ng/mL serum cortisol levels (assuming that serum specimens could be directly applied by diluting tenfold). Considering the normal levels for serum cortisol (10–250 ng/mL)^30^, these ELISA systems are sensitive enough for diagnostic applications. Although cross-reactivity with eight kinds of endogenous steroids indicated that the amino acid substitution accompanied alteration in specificity (Supplementary Table S1), the recognition pattern of these affinity-matured scFvs was, overall, acceptable from the view of clinical application^16,19^.

### Application of CAP to a different library composed of FR1-extended scFvs

**Figure 6:**
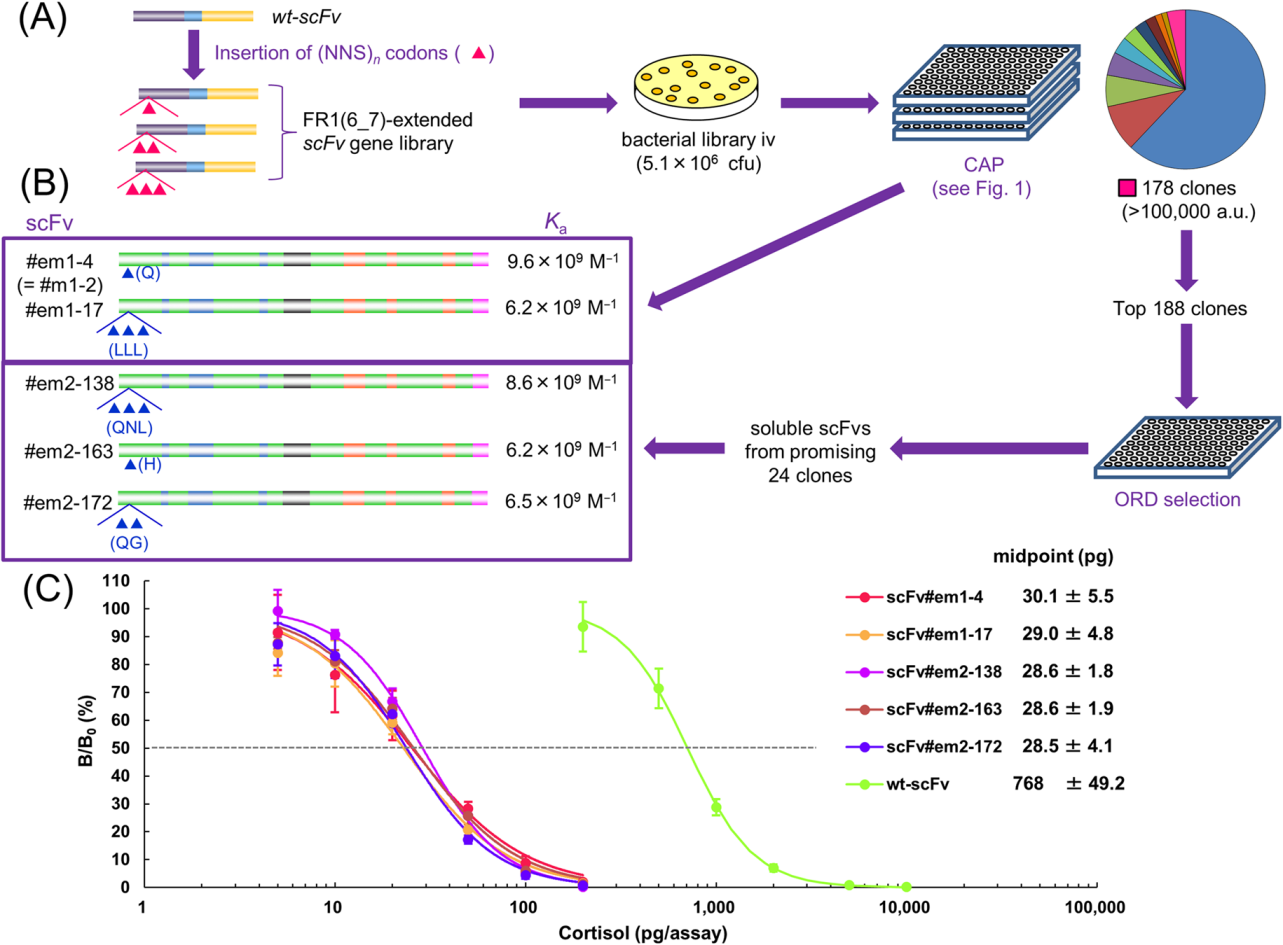
CAP-based discovery of affinity-matured mutants from the FR1-extended scFv library and their performance in ELISA. **(A)** scFv mutants with improved affinities were isolated with CAP from a library of anti-cortisol scFv each having an extra amino acid(s) inserted in a site-directed manner. Between the codons encoding Q at the V_H_6 and P at the V_H_7, located in the V_H_-FR1 of wt-scFv, the (NNS)_*n*_ degenerated codon(s) (*n* = 1, 2, or 3) were inserted by PCR using synthetic oligo-DNAs. The *E. coli* TG1 cells were transformed with the resulting *scFv* mutant genes. A small portion of the resulting bacterial library iv was submitted to CAP as illustrated. **(B)** Among them, scFv#em1-4 and 17 found without ORD selection and scFv#em2-138, 163, and 172 found with ORD selection showed > 16-fold increased *K*_a_ versus that of wt-scFv. The arrowhead(s) denotes insertion(s) of amino acid(s), also shown in the one letter code. scFv#em1-4, #em2-138, and 172 were the product by readthrough of an amber codon inserted directly after the V_H_6-codon, and estimated to be decoded as Q similarly to the case of scFv#m1-2. **(C)** Typical dose–response curves for cortisol in competitive ELISAs using wt-scFv and the improved scFvs. The vertical bars indicate the SD for intra-assay variance (*n* = 4). The midpoint values (mean ± SD for inter-assay variance; *n* = 4) are listed together. The ELISA conditions were controlled as described in Fig. 5.

In the initial trial of CAP described above, we obtained an affinity-matured scFv with a rare and suggestive structural alteration; namely, scFv#m1-2 that showed ~ 10^10^ M^–1^ affinity owing to the insertion of an extra amino acid Q between the amino acids at the position 6 (Q) and 7 (proline; P), located in the FR1 of V_H_ domain (Fig. 4A). This finding prompted us to generate a new library of anti-cortisol scFvs having extended FR1 sequences due to the insertion of a single or, doubly- or triply-consecutive randomized amino acid(s) between the positions V_H_6 and V_H_7 (Fig. 6A). This library theoretically includes 8,420 (= 20 + 20^2^ + 20^3^) different sequences. We submitted a portion (~ 0.1%) of the bacterial library transformed with the resulting *scFv* genes to CAP without and with ORD selection (Fig. 6A). Only two operations provided five scFv mutants showing > 16-fold increased *K*_a_ values (Fig. 6B). We should emphasize that the same clone as scFv#m1-2 (named scFv#em1-4 here), which should be contained in the new library, was isolated again. These mutants, when used for ELISA, showed obviously higher sensitivity (midpoint ranged 28–30 pg/assay; meaning 26–27-fold improvement) than that in the ELISA using wt-scFv (Fig. 6C).

## Discussion

It is well known that the phage display-based antibody engineering (i.e., “antibody-breeding”) strategy has supplied precious antibody reagents, both for therapeutic and diagnostic use^1–8^. However, the panning, which should work as a key process therein, has often troubled us with its inefficiency and uncertainty due to somewhat fluctuating reproducibility as shown with the present study (Fig. 3B) in selecting improved species. To radically avoid such panning-inherent problems, we constructed CAP method, in which colonies of the initial bacterial libraries are examined one-by-one in individual microwells, so as not to disturb their original diversity and not to compete with different clones during bacterial propagation and following events. Monoclonal scFv-phage progenies should be generated in the microwell where the parent bacterium was inoculated and propagated, maintaining the genotype (*scFv* gene in the bacterium) and phenotype (scFv protein displayed on phage) linkage. If scFv-phage clones show significant antigen-binding ability, they should be captured in the microwell via a pre-coated antigen, forming microwell-based array of antigen-binding scFv-phage clones, which are then detected in a sensitive bioluminescent assay with the aid of the anti-M13-GLuc fusion protein. To improve the efficiency in obtaining high-affinity binders, an advanced variation of the profiling involving “ORD selection” was also devised.

We should refer, here, to the selection based on next-generation sequencing (NGS), which attracted much attention several years ago. NGS has enabled the ranking of antibody-displaying phage clones enriched after the panning, based on the frequency of gene sequence encoding a part of the variable regions^31–34^. Lesser antigen-binding dependency makes this strategy advantageous when target antigens are difficult to obtain. However, due to the panning-inherent problem described previously, more frequent sequences do not always associate with more improved mutants in terms of antigen-binding characteristics. To confirm whether the phenotype of the selected genes in fact exhibits improved antigen-binding abilities, considerable effort is required for constructing the genes encoding the full-length antibody fragments anew and for their expression.

The CAP approach that we have proposed here might be regarded as a return to “primitive-in-a-sense” screening from “theoretically-rational and elegant” selection (panning). However, we are convinced that CAP should be the most straightforward and robust way to overcome the selection problem, and should enable high-throughput isolation of improved species. It might be worth referring to that, in the middle of 1990s, the Stemmer’s group suggested the efficiency of individual screening of ~ 10,000 transformants for finding improved mutants in their study for generating improved green fluorescent protein species^35^. In fact, an advantage was evidenced by its application to a small, easily-preparable randomly-mutated library of anti-cortisol scFvs. Even though only two operations (with or without the ORD selection step) were performed each on a small portion (only ~ 3%) of the library, we obtained eight mutants showing > 14-fold improved *K*_a_ values over the wild-type scFv, and five of the eight exhibited 10^10^-range *K*_a_ values. In contrast, ten operations of conventional panning on the entire library (i.e., 100% of the colonies), even with the three different protocols, only provided seven moderately improved binders (with 1.1–5.0-fold higher *K*_a_). The fact that the panning protocol (III) provided no improved clone, despite the previous contribution for isolating an improved scFv against cortisol derived from the same wild-type scFv as that used in this study (Fig. 2C)^16^, demonstrated difficulty in panning-based selections.

The utility of CAP was further supported by the additional trial mining of a different library that was composed of scFvs with extended FR1 sequences, from which five improved mutants (with > 16-fold improved *K*_a_ values) were successfully isolated. As we discussed previously^10,14^, improving antibodies against low-molecular-weight antigens (haptens) such as cortisol, dealt with in this study, is challenging. Particularly, mining affinity-matured mutants can become dramatically difficult when the parental scFv already shows significant affinity (e.g., *K*_a_ > 10^8^ M^−1^) for the target antigen, like our instances shown in Fig. 2. During the period of about a quarter century after in vitro evolution was established, less than 10 studies have succeeded in practical affinity maturation: in which mutants with a *K*_a_ of > 10^9^ M^−1^ were generated after over fourfold improvement, and the mutants were reactive against free (not immobilized) hapten molecules^10,14^ (Supplementary Fig. S6). The CAP method, however, facilitated success by generating a total of 13 mutants that exceeded this criterion (Figs. 4, 6), even with only four operations (counting the mining of FR1-extended library together), each working with a randomly selected small portion (9,400 or 4,700 colonies) of a small library (containing 10^5^ ~ 10^6^-members as cfu) as summarized in Figs. 3 and 6. Extensive working power that is required for performing such a screening-based approach will be diminished in the future by automatization using robotic colony pickers so as to allow for full screening of > 10^6^-size libraries. If we could process the entire library discussed above, we should, mathematically, obtain more similarly-improved mutants, i.e., 267 [= 8 × (100/3)] from the randomly-mutated library (Fig. 3C or C’) and 5,000 [= 5 × (100/0.1)] from the FR1-extended library (Fig. 6A). Analytical significance of the CAP-derived mutant scFvs was shown in the competitive ELISA, dose–response curves of which were obviously more sensitive than that with wt-scFv (Figs. 5 and 6C).

During this study, we obtained some unexpected and important information regarding the primary structures of the affinity-matured mutants. The CAP-derived mutants from the randomly-mutated library had prominently different features from those obtained with the panning (Fig. 4A), represented by the less substitutions (and insertions) that were concentrated more in the V_H_ and in CDRs. It should be noted that no scFv mutant obtained with CAP, as well as that recovered from the panning, had substitution/insertion in the V_H_-CDR3 that is regarded as the most crucial among the six CDRs to generate affinity and specificity against a definite antigen structure^11^(Fig. 4A). Moreover, the substitutions/insertions frequently occurred in FR1 in the V_H_ domain, suggesting that this region might be an “untapped hotspot,” where mutations introduced therein might result in the affinity maturation in high probability. This assumption was strongly supported by our additional application of CAP to the FR1-extended scFv library (Fig. 6). The FR1 is unlikely to participate for direct contact with the antigens, as suggested by the protein modelling of scFvs (Fig. 4B), but might interact with the loop structure of V_H_-CDR3 due to the proximity. Otherwise, it might influence the dimerization or thermodynamic stability of scFvs^36^. In addition, isolation of affinity-matured mutant with a single substitution on the linker (scFv#m1-18) was remarkable, and prompts us to generate a small library by randomization of the linker sequence.

One more interesting feature was the role of “unusual” C (cysteine) at the position 49 in the V_L_ domain referred above. While the panning-derived and moderately improved mutants all deleted this C by substitution, the CAP-derived and successfully improved mutants all retained this residue. Therefore, it is reasonable to deduce that this C plays an important role for exerting the cortisol-binding affinity. This was supported by additional experiments, in which we examined the affinities (estimated with ELISA dose–response curves) of 10 kinds of V_L_97-substituted mutants prepared from scFv#m2-97 (the mutant with the highest *K*_a_ in this study) (Supplementary Fig. S7). Isoleucine (I)- and tyrosine (Y)-substituted mutants showed a slightly-improved response over scFv#m2-97, however, the other eight mutants all deteriorated significantly.

Our CAP might be associated with colony-lift assays, which were developed in the 1990s^37–40^. This method arrowed direct and simultaneous screening for multiple bacterial colonies regarding the ability to produce antigen-binding scFv (or Fab), but remained several problems that hamper reliable discovery of affinity-matured mutants: i.e., insufficiency of reproducibility, quantitativity, resolution between colonies growing closely on agar. Moreover, the phenotype (visualized spots) and genotype (causal bacterial colonies) were separated on different membranes. We overcame these drawbacks using a “one-pot” (i.e., same microwell) and individual operation for cultivating monoclonal transformants and monitoring the propagated scFv-phages with robust immunoreactions. To enable this, proliferation potency of monoclonal phages and high sensitivity of the bioluminescent assay were mandatory. Therefore, CAP works successfully by taking advantage of the intrinsic advantage of phages; i.e., the potential of replication, similar to the panning where the propagating nature is also essential for multiplying trace amounts of recovered phages for the following stage of work. The analysis of individual scFv-phage particles using a flow cytometry (FC)-like principle seems to be a more sophisticated approach. This might be realized simply by adding FC-applicable microbeads coated with CS-BSA one-by-one into the microwells, in which individual transformant grows and generates scFv-phages. It should be noted that a similar and elegant principle has been reported that used micro droplets in water-in-oil emulsions as the compartments that correspond to our microwells^41^.

In conclusion, the present CAP should readily be applicable for laboratories with standard equipment, and should overcome the longstanding trouble in isolating rare and improved antibody species, enabling the isolation of conventionally-overlooked mutants. CAP will contribute widely for providing “next-generation” antibody reagents useful for both therapeutic and diagnostic applications. We have already undertaken modification of the present CAP into more efficient and feasible procedures. To surely recover scFv mutants bound on the antigen-immobilized solid phases with extremely high affinity, we might employ the dissociation-independent methods such that we previously reported^14,42,43^.

## Materials and methods

### Buffers

PB: 50 mM sodium phosphate buffer (pH 7.3); PBS: PB containing 9.0 g/L NaCl; G-PBS: PBS containing 1.0 g/L gelatin; T-PBS: PBS containing 0.050% (v/v) Tween 20; M-PBS: PBS containing 20 g/L skim milk; PVG-PBS: G-PBS containing 1.0 g/L polyvinyl alcohol (average polymerization degree 500; Nacalai Tesque); PBS-2: 10 mM Na_2_HPO_4_, 1.8 mM KH_2_PO_4_, 0.14 M NaCl, 2.7 mM KCl (pH 7.4); M-PBS-2: PBS-2 containing 20 g/L skim milk; and T-PBS-2: PBS-2 containing 0.10% (v/v) Tween 20^12–16^.

### Production of anti-M13-GLuc

A gene encoding the scFv derived from Ab-M13#71^18^ was amplified using the M13#71V_H_-Rev^18^ and CS#10V_L_-For-2^19^ primers to add another linker sequence at the 3′-end for fusion with the *GLuc* gene (a gene fragment encoding “wild-type” *Gaussia* luciferase^19^ followed by sequences encoding FLAG and His6 tags, and TAATGA codons) (Supplementary Fig. S2). The re-amplified *scFv* and *GLuc* gene fragments (200 ng each) were spliced by performing overlap-extension PCR^12–16^. The resulting *anti-M13-GLuc* fusion gene was expressed in *E.coli* XL1-Blue cells (Agilent Technologies) under induction with isopropyl β-D-thiogalactopyranoside and sucrose^12–16^. Periplasmic extracts containing anti-M13-GLuc fusion protein was used for phage detection without further purification.

### Bioluminescent detection of scFv-phages bound to microwells

White 96-well microplates (#3922; Corning) were coated with BSA conjugated with cortisol^19^ or estradiol-17β^12–14^, using 1.0 µg/mL solutions in 0.10 M carbonate buffer (pH 8.6), and blocked with Block Ace (DS Pharma Biomedical). scFv-phages against cortisol or estradiol-17β [0–10^9^ colony-forming units (cfu)], rescued with KM13 helper phage^17^, were added as suspension in 2 × YT medium to the microwells (200 µL/well) and incubated at 37 ºC for 60 min. After washing with T-PBS-2 three times, appropriately diluted anti-M13-GLuc in M-PBS was added (100 µL/well) and further incubated for 30 min at 37 ºC. After washing, 5.0 µM coelenterazine (Nanolight) in PBS was added (100 µL/well), mixed, and scanning (2.4 well/s) of luminescence (emission at a range of 300–700 nm was collected) was initiated 27 s after addition of the substrate using a Synergy HTX multi-mode microplate reader (Biotech).

### Construction of the randomly-mutated anti-cortisol scFv gene library

Error-prone PCR^12–16,20^ was performed to amplify the anti-cortisol *wt-scFv* gene^16^ in a buffer solution (100 µL) with 0.10 mM MnCl_2_, the reverse (5′-GGATTGTTATTACTCGCGGC-3′) and forward (5′-CCTGATGTGTG CGTCTTAGT-3′) primers (0.10 nmol each), AmpliTaq DNA polymerase (Applied Biosystems) (5 U), and 0.10 µmol of each dNTP (except dATP, which was present at 0.020 µmol). These mixtures were amplified for 35 cycles of 95 ºC (1 min), 50 ºC (1 min), and 72 ºC (3 min), followed by a 10-min extension at 72 ºC. The mutated *scFv* genes were ligated into the pEXmide 7 vector^16^. TG1 cells (Agilent Technologies) were transformed with the resulting plasmid (*ca*. 0.4 µg) by electroporation. After an electrical pulse was discharged, the cells were immediately incubated in SOC medium (1.0 mL) at 37 °C for 60 min. For the conventional panning, the resulting cell suspension was centrifuged (1,800 × *g*, 20 min), the pellet was suspended in SOC medium (100 µL), and spread on 90φ plates containing 2 × YT agar supplemented with 100 µg/mL ampicillin and 1.0% glucose. The plates were incubated overnight at 37 ºC and all the colonies grown were scraped into a 2 × YT medium containing 15% glycerol (1.5 mL). The resulting suspension was divided into aliquots and stored at – 80 ºC, ten of which were used for the panning later. For CAP, the aforementioned cell suspension in SOC medium was adequately diluted with the SOC medium, aliquots (200 µL) of which were then spread on 144 × 100 mm rectangle plates containing 2 × YT agar supplemented as above. After overnight incubation at 37 ºC, the colonies grown were subjected to CAP as described later.

### Phage rescue and the conventional panning

The aliquots of the ten glycerol stocks for the bacterial library transformed with the randomly-mutated anti-cortisol *scFv* genes were each inoculated into 2 × YT medium containing 100 µg/mL ampicillin and 2.0% glucose (50 mL), and shaken (200 rpm) at 37 ºC until the absorbance (600 nm) reached ~ 0.4. The KM13 helper phage was added at a multiplicity of infection of 20, and the mixture was incubated at 37 ºC for 30 min. After centrifugation (1,000 × *g*, 20 min), the precipitated cells were resuspended in 2 × YT medium containing 100 µg/mL ampicillin and 50 µg/mL kanamycin (20 mL × 2), and then shaken (120 rpm) for 12–16 h at 25 ºC. This culture was centrifuged and the virions generated were precipitated by adding polyethylene glycol 8,000 in 2.5 M NaCl (5.0 mL). After centrifugation, the precipitated phages were resuspended in sterile PBS-2 and submitted to one of the three panning experiments described below. In these panning protocols (I)–(III), in common, three rounds of selections were performed using MaxiSorp 12 × 75 mm polystyrene tubes (ThermoFisher Scientific) (i.e., immunotubes) coated with solutions of CS-BSA in the carbonate buffer^19^ and blocked with M-PBS-2^13^. Therein, ~ 1 × 10^11^ cfu/tube phages were input as a suspension in PVG-PBS (2.0 mL) and incubated for 60 min at 37 ºC with continuous tumbling.

*Protocol (I)*: The immunotubes were coated in sextuplicate with 5.0 µg/mL solution of CS-BSA (2 mL/tube). After the incubation with phages, the tubes were washed three times with T-PBS-2 (4 mL), and bound phages were eluted by incubation with 0.10 M glycine-HCl (pH 2.2) or 0.10 M triethylamine (pH 12) (1.0 mL) at room temperature for 10 min. The solutions recovered were neutralized by addition of 2.0 M Tris (pH 10.6) or 1.0 M Tris–HCl (pH 7.4), respectively. *Protocol (II)*: The immunotubes were coated in duplicate with 5 µg/mL, 0.5 µg/mL, or 0.05 µg/mL solution of CS-BSA (2 mL/tube) for the first, second, or third round, respectively. After the incubation with phages, the tubes were washed three, six, or twelve times with T-PBS-2 (4 mL) for the first, second, and third round, respectively, and the bound phages were eluted by incubation with 0.10 M glycine-HCl (pH 2.2) (1.0 mL) at room temperature for 10 min. The solutions recovered were neutralized as above. *Protocol (III):* The immunotubes were coated in duplicate with 0.5 µg/mL solution of CS-BSA (2 mL/tube). After the incubation with phages, the tubes were washed three, three, or five times with T-PBS-2 (4 mL) for the first, second, and third round, and the bound phages were eluted by incubation with 10, 1, or 0.1 ng/mL solution of cortisol in PVG-PBS (1 mL/tube) for 120 min at 37 ºC.

The eluted phages were added to log-phase TG1 cells in 2 × YT medium (9 mL), and then incubated at 37 ºC for 30 min. After centrifugation (10,000 × *g*, 10 min), the pellet was resuspended in 2 × YT medium (100 µL) and spread on 90φ plates containing 2 × YT agar supplemented with 100 µg/mL ampicillin and 1.0% glucose, and incubated overnight at 37 ºC. The colonies were then scraped into 2 × YT medium containing 15% glycerol (1.5 mL) and an aliquot was used for phage rescue in the next round of selection^12–16^.

### CAP

Individual bacterial colonies grown on agar (described above) were picked with sterile toothpicks and dipped in 2 × YT liquid medium containing 100 µg/mL ampicillin, 5.0 µg/mL kanamycin, and *ca*. 5 × 10^8^ pfu/mL KM13 helper phage, which was filled in microwells (200 µL/well) of 96-well white microplates (#3922) pre-coated with CS-BSA as described above. After incubation at 25 ºC for 45 h with continuous shaking (800 rpm), these microplates were washed three times with T-PBS-2, and appropriately diluted anti-M13-GLuc in M-PBS (100 µL/well) was added, and the plates were incubated at 37 ºC for 30 min. After washing, 5.0 µM coelenterazine solution was added (100 µL/well), mixed, and the luminescence was scanned as described above (Fig. 1D). For microwells that generated strong luminescence, the mixture therein was removed, 0.10 M glycine-HCl (pH 2.2) was added (100 µL/well), and incubated at room temperature for 10 min. The solutions recovered were neutralized as above and the scFv-phages contained therein were propagated through infection to TG1 cells in log-phase growth for characterization.

### ORD selection of scFv-phages

The scFv-phages that showed high luminescence in CAP were recovered and added to liquid 2 × YT medium containing *ca*. 3 × 10^8^ cells/mL TG1 cells growing in log phase (100 µL/well) in microplates (#3922) coated with CS-BSA. After incubation at 37 ºC for 30 min, 2 × YT medium containing 200 µg/mL ampicillin, 10 µg/mL kanamycin, and 1 × 10^9^ pfu/mL KM13 helper phage was added (100 µL/well) and the microplates were incubated at 25 ºC for 45 h with continuous shaking (800 rpm). Then, the microplates were washed, reacted with anti-M13-GLuc, the coelenterazine solution was added, and scanned to read the luminescence of each well as described above to obtain “initial signals”. The microplates were washed again, and 5.0 µg/mL (14 µM) cortisol dissolved in G-PBS (200 µL/well) was incubated therein at 25 ºC for 4.0 h. After removing the mixture and washing, the luminescence of each well was read again. This cycle was repeated three more times and the decreasing luminescence due to progressively diminishing bound scFv-phages was monitored. scFv-phages that still showed relatively high luminescence were propagated through infection to TG1 cells for characterization.

### Preparation and characterization of soluble scFvs

scFv-phages selected as described above were transformed into the corresponding soluble (non-phage-linked) scFv proteins according to our previous method^16^, and their analytical performance in the competitive ELISA was examined. The 96-well microplates (#3590; Corning) coated with CS-BSA were incubated at 4 ºC for 120 min with a mixture of cortisol (or analogous steroid) standard (50.0 µL/well) and soluble scFv protein (100 µL/well), both diluted in G-PBS. The microplates were washed three times with T-PBS and probed using an anti-FLAG M2 antibody labeled with peroxidase (POD) (Sigma–Aldrich), diluted in G-PBS (0.20 µg/mL; 100 µL/well)^12–16^. After incubation at 37 ºC for 30 min, the microplates were washed and the captured POD activity was colorimetrically determined (490 nm) employing *o*-phenylenediamine as chromogen^12–16^. To construct the ELISA dose–response curves, GraphPad Prism version 3.0a (GraphPad Software) was used for curve fitting and determination of the reaction parameters. The midpoint (i.e., IC_50_) values were derived from a four parametric logistic equation [log(analyte dose) vs. B/B_0_ (%)] as the EC50 value. The top (the maximum response) and bottom (the basal response) were constrained to constant values of 100 and 0, respectively. The unit "X g/assay" was used in the abscissa, which means that a total of X g (mass) of the analyte or cross-reactive analogs was added to the assay chamber (microwells) for the competitive antigen–antibody reactions.

### Determination of affinity parameters for soluble scFvs

The equilibrium affinity constants (*K*_a_) of scFvs were determined by the Scatchard analysis^21^. Mixtures of [1, 2, 6, 7-^3^H]-cortisol (3.53 TBq/mmol; PerkinElmer) (*ca*. 250 Bq), varying amounts of standard cortisol, and a constant amount of each scFv (adjusted to capture *ca*. 50% of the tritium-labeled cortisol in the absence of standard cortisol) were incubated in GPB (700 µL) at 4 ºC overnight (~ 16 h). The bound (B) and free (F) fractions were separated by a dextran-coated charcoal method, and radioactivity of the B fraction was measured. Dissociation rate constants (*k*_d_) of the soluble scFvs obtained by the ORD selection were determined by the BLI^24^ at 25 °C using BLItz (ForteBio), a BLI sensor. Streptavidin-coated biosensor tips, which were saturated with a biotin-labeled CS-BSA, prepared by reaction with EZ-Link NHS-LC-Biotin (Thermo Fisher Scientific)^44,45^, were dipped into 4.0-µL scFv solutions in G-PBS (50–1,600 nmol/L). Association of the scFvs with the cortisol residues was monitored for 120 s, and then dissociation was measured for 600 s in G-PBS.

### Preparation and CAP-based selection of the anti-cortisol FR1-extended scFv library

PCR was performed to amplify the anti-cortisol *wt-scFv* gene subcloned in pEXmide 7 vector^16^ in a buffer solution (100 µL) with *KOD Fx* DNA polymerase (TOYOBO) (5 U), 40 nmol of each dNTP, using one of the following reverse primers 5′-NNS1 (5′-ATTGTTATTACTCGCGGCCCAACCGGCCATGGCCCAGGTCCAACTGCAGCAGNNSCCTGGGGCTGAACTTGTGAAGC), 5′-NNS2 (5′-ATTGTTATTACTCGCGGCCCAACCGGCCATGGCCCAGGTCCAACTGCAGCAGNNSNNSCCTGGGGCTGAACTTGTGAAGC), or 5′-NNS3 (5′-ATTGTTATTACTCGCGGCCCAACCGGCCATGGCCCAGGTCCAACTGCAGCAGNNSNNSNNSCCTGGGGCTGAACTTGTGAAGC) in combination with the fixed 3′-primer, CS#3V_L_-For^16^ (50 pmol each). These mixtures were amplified for 94 ºC (2 min) followed by 35 cycles of 98 ºC (10 s), 55 ºC (30 s), and 68 ºC (1 min). The resulting three kinds of *scFv* mutant genes were separately ligated into the pEXmide 7 vector^16^. Then the reaction mixtures were equally mixed, with which TG1 cells were transformed by electroporation. After an electrical pulse was discharged, the cells were immediately incubated in SOC medium (1.0 mL) at 37 °C for 60 min. The cell suspension was adequately diluted, spread on the rectangle agar plates, and colonies grown were subjected to CAP as described above.

## Supplementary information

Supplementary Information.

## Data Availability

The data sets generated during the current study are available from the corresponding author upon request.
