# Supplementary material for: Clonal array profiling of scFv-displaying phages for high-throughput discovery of affinity-matured antibody mutants
Source: Sci Rep. 2020 Aug 24;10:14103. doi: 10.1038/s41598-020-71037-3 (PMC7445280; doi:10.1038/s41598-020-71037-3)
Supplement: Supplementary file 1 — Supplementary Information. [file 41598_2020_71037_MOESM1_ESM.pdf]

Supplementary Information

Clonal array profiling of scFv-displaying phages for high-throughput discovery of affinity-matured antibody mutants

Yuki Kiguchi, Hiroyuki Oyama, Izumi Morita, Mai Morikawa, Asuka Nakano, Wakana Fujihara, Yukari Inoue, Megumi Sasaki, Yuki Saijo, Yuki Kanemoto, Kaho Murayama, Yuki Baba, Atsuko Takeuchi, and Norihiro Kobayashi*

Kobe Pharmaceutical University, 4-19-1, Motoyama-Kitamachi, Higashinada-ku, Kobe 658-8558, Japan

Methods

LC/MS/MS fingerprinting for scFv#m1-10 to identify the amino acid incorporated due to nonsense suppression. The soluble form scFv#m1-10 protein was run on polyacrylamide gel, and the gel slice containing the protein was suspended in Tris-HCl buffer (*ca*. 100 μg/mL) and submitted to trypsin digestion using commercially available kits (XL-Tryp kit; Apro Science). LC/MS/MS was performed on an LTQ-Orbitrap Discovery (linear ion trap–orbitrap) spectrometer (Thermo Fisher Scientific), which was connected to a Dionex UltiMate 3000 pump and a HTC-PAL auto-sampler (CTC Analytics). The mobile phases consisted of 0.10% formic acid in water (solvent A) and 0.10% formic acid in acetonitrile (solvent B). A solution of the scFv-derived peptides, dissolved in 0.10% trifluoroacetic acid (35 μL), was applied to the LC/MS/MS system. The peptides were fractionated on an L-column Micro C-18 (150 mm length × 0.10 mm diameter; particle size, 3 μm; Chemicals Evaluation and Research Institute) with a linear gradient of 3.0–43% solvent B for 40 min at a flow rate of 500 nL/min. The column eluent was sprayed directly into the ion source of the mass spectrometer, using a spray tip (Fortis tip, AMR) with a spray voltage of 1.8 kV. The "lock mass" function was used to obtain high mass accuracy during the fractionation. The mass spectra were measured in a range of 300–2000 *m*/*z* ratio. In each mass spectrum of eluents, the top seven high-intensity precursor ions were selected automatically for subsequent product ion analysis by a data-dependent scan mode with a dynamic exclusion option. The LC/MS/MS data were interpreted using Proteome Discoverer (Thermo Fisher Scientific). Peptides were identified from the self-made database, with a peptide mass tolerance of 4 ppm and a fragment mass tolerance of 0.80 Da.

Preparation and characterization of V_L49-substituted mutants of scFv#m2-97. PCR was performed to amplify the anti-cortisol *scFv#m2-97* gene subcloned in pEXmide 7 vector[1] in a buffer solution (100 μL) with *KOD Fx* DNA polymerase (1 U), 40 nmol of each dNTP, using one of the following six kinds of reverse primes CS3VL-49C(-)-1–6: 5'-CCACCCAAACTCCTCATC<u>XXX</u>CTTGCATCCAGCCTAGAA [<u>XXX</u> = GGC(1), AGC(2), TGG(3), TWT(4), AHC(5), or CKC(6)] in combination with the fixed 3'-primer, CS#3V_L-For[1] (50 pmol each). These mixtures were amplified for 94ºC (2 min) followed by 40 cycles of 98ºC (10 sec) and 68ºC (1 min). Then, the *scFv#m2-97* gene was amplified again using one of the resulting products as 3'-megaprimer (*ca*. 350 ng) in the combination with CS#3V_H-Rev[1] (50 pmol) under the conditions of 94ºC (2 min) followed by 40 cycles of 98ºC (10 sec) and 60ºC (1 min). The amplified products containing full-length

scFv genes were each gel-purified, digested with *Nco*I and *Not*I, and ligated into the pEXmide 7' vector[1]. *E. coli* XL1-Blue cells were transformed with the resulting plasmid (*ca.* 0.2 μg) by electroporation, and the single transformant was grown for preparing soluble form scFv proteins as the periplasmic extracts as described in the text. The scFvs obtained were submitted to the ELISA (the procedure was described in the text) without further purification to estimate their affinity based on the midpoint of dose–response curves (Supplementary Fig. S7).

Figures

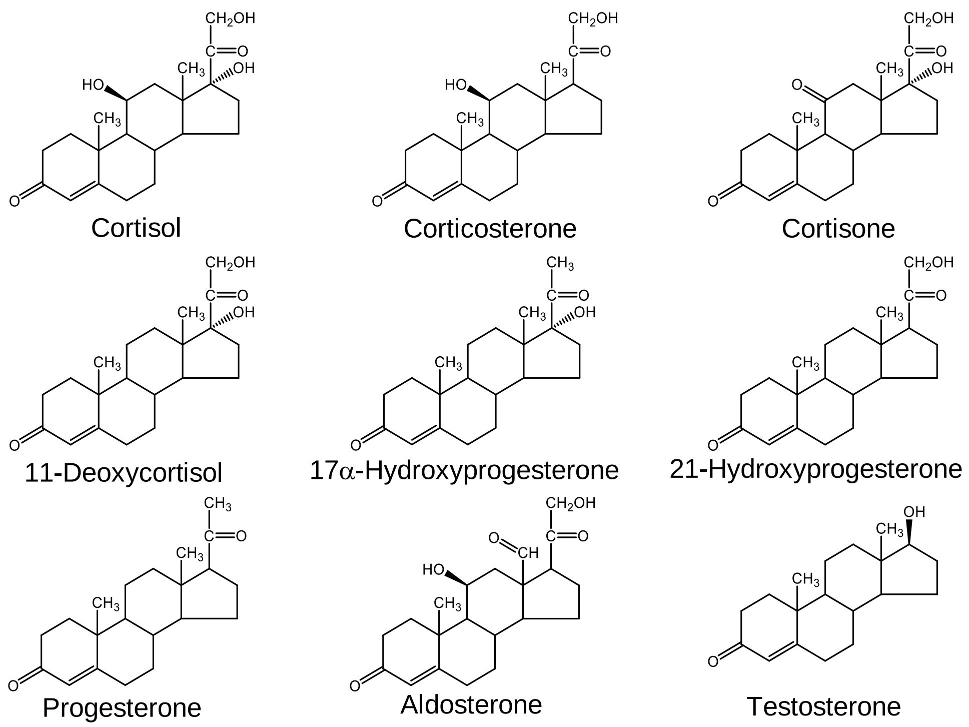

Fig. S1. Chemical structures of endogenous steroids that are referred to in this study.

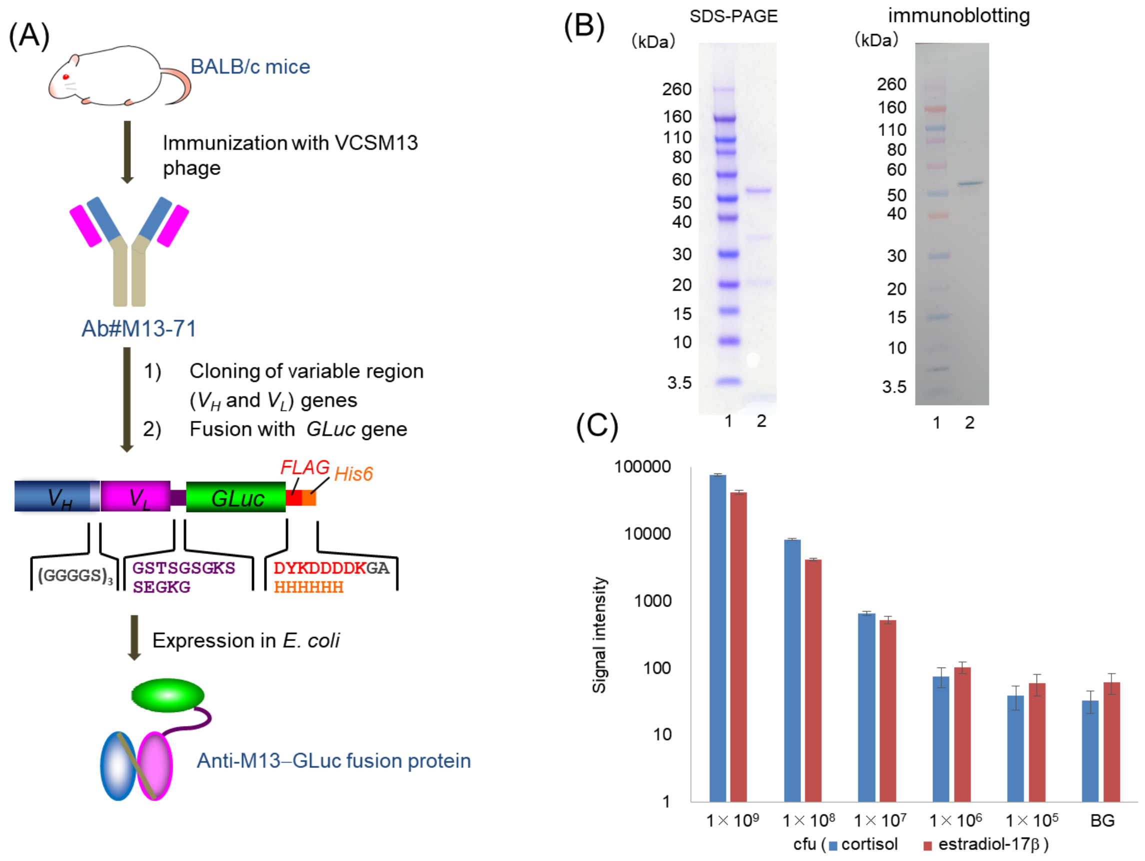

Fig. S2. Generation and characterization of anti-M13–GLuc fusion protein. (A) A mouse monoclonal antibody Ab-M13#71 (IgG2b, κ), specific to the major coat protein pVIII of the VCSM13 phage, was converted to the scFv form[2], in which the V_H and V_L domains were combined via linker1, *i.e.*, the common $(GGGGS)_3$ sequence[3,4]. The corresponding *scFv* gene was fused with a wild-type *GLuc* gene[5] via a peptidase-resistant linker (GSTSGSGKSSEGKG; linker2)[6,7] in the orientation of 5'-V_H-*linker1*-V_L-*linker2-GLuc-FLAG-His6-TAATGA*, which was expressed in the *E. coli* XL1-Blue cells. (B) Sodium dodecyl sulfate-polyacrylamide gel electrophoresis (SDS-PAGE) analysis (Coomassie brilliant blue staining) (left) and immunoblotting (right) of the anti-M13–GLuc fusion protein after purification by affinity chromatography using anti-FLAG-M2 agarose (Sigma–Aldrich): lane 1, *Mr* marker; 2, anti-M13–GLuc fusion protein. These pictures were excerpted from the original pictures that contained the bands for an unrelated sample (shown below in Fig. S2'). In the immunoblotting, proteins separated were transferred to a PVDF membrane and blocked with M-PBS. The fusion protein was detected with the serial reactions with VCSM13 helper phage (1×10^{12} pfu; Agilent Technologies) and POD-labeled anti-M13 antibody (GE Healthcare). Bound POD activity was visualized with hydrogen peroxide and 3,3',5,5'-tetramethylbenzidine. The product resolved at the calculated relative molecular mass (M_r) (48292; including linkers and tag peptides) and the blotting experiments indicated to be expressed in-frame to the end of the C-terminus. (C) Detection of scFv–phages against estradiol-17β[8,9] and cortisol[5] captured on antigen-immobilized microplates with the anti-M13–GLuc. For both scFv–phages, $1{\times}10^6$ cfu virions were detected ($P < 0.05$). The vertical bars indicate the standard deviation ($n = 4$).

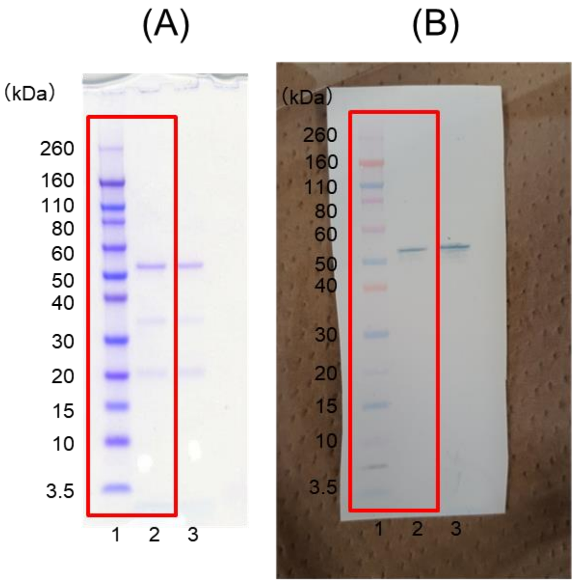

Fig. S2'. The original pictures of (A) the whole gel of SDS-PAGE analysis and (B) the whole membrane for the immunoblotting of the affinity-purified anti-M13–GLuc fusion protein: lane 1, *Mr* marker; 2, anti-M13–GLuc fusion protein, and lane 3, a fusion protein combining the anti-M13 scFv with a GLuc mutant (scFv–mGLuc1)[5]. The areas shown with red rectangles were used in Fig. S2(B).

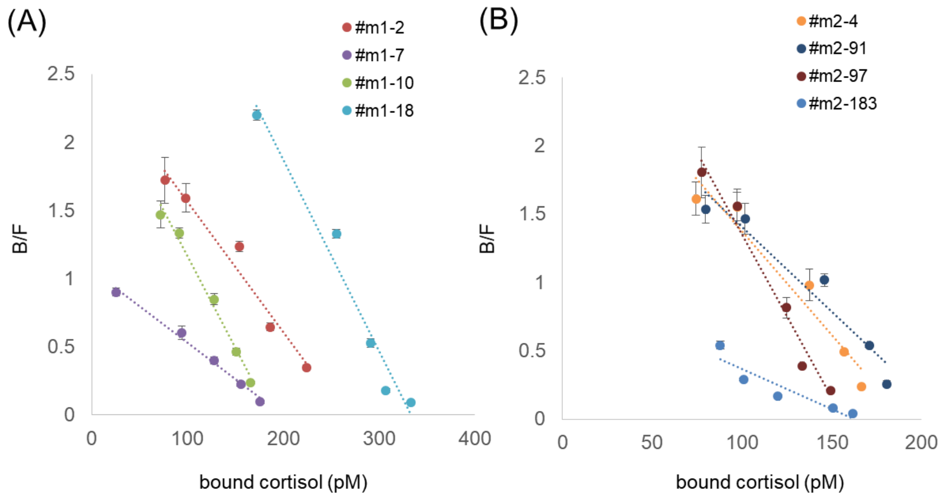

Fig. S3. Scatchard analysis for soluble scFvs to determine K_a values. The results of Scatchard analysis[10] for eight kinds of scFv mutants, (A) scFv#m1-2, 7, 10, and 18 obtained from CAP without off-rate-dependent (ORD) selection and (B) scFv#m2-4, 91, 97, and 183 from CAP with ORD selection, are shown. The vertical bars indicate the standard deviations (n = 4).

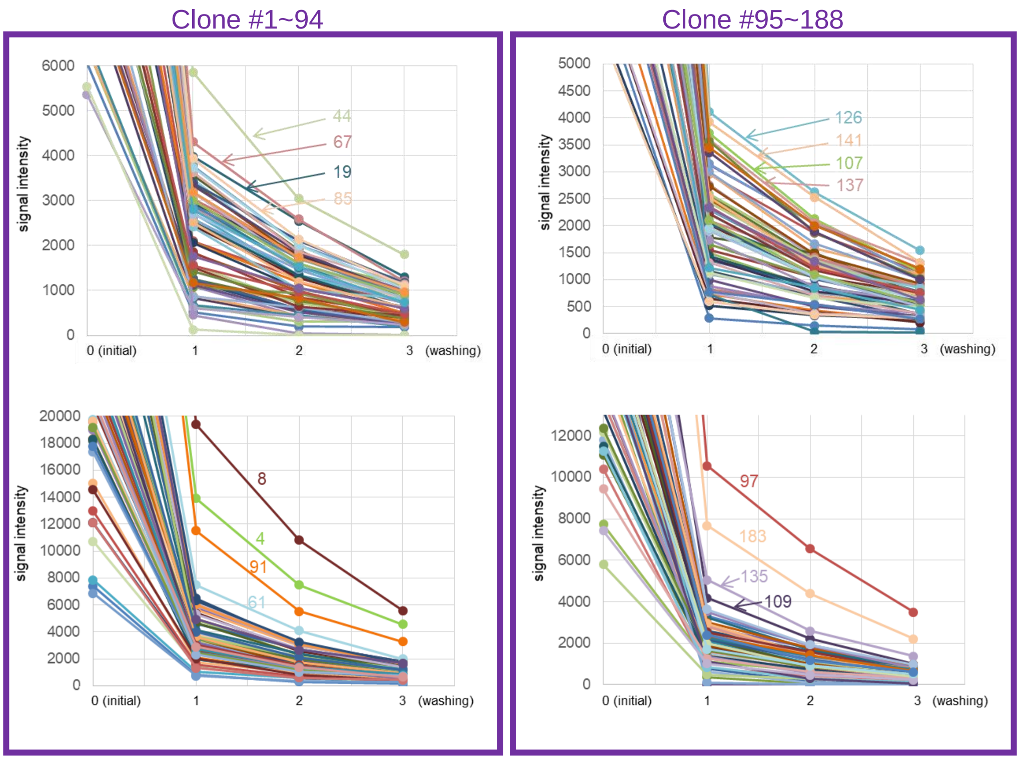

Fig. S4. Monitoring of the decrease in scFv–phages bound to microplates during ORD selection. ORD selection was performed for the 376 scFv–phages that showed strong luminescence during the initial CAP. The bioluminescent assay was performed four times: *i.e.*, to determine “initial binding” and after each of the three incubation periods with a solution of free cortisol to remove scFv–phages with higher off-rates. In these incubation steps, we set the molar ratio of the free cortisol added versus the immobilized cortisol-residues to be 300:1 to prevent re-binding of dissociated scFvs to the immobilized cortisol residues. The incubation was performed for 4 hours, which is near the $t_{1/2}$ of scFvs with the k_d of *ca*. 5 × 10^{-5} s^{-1}. The 16 scFv–phages (indicated in the figure) that showed a more gradual decrease in the

luminescence (showing >1,000 a.u. luminescence in the fourth bioluminescent assay; clones #4, 8, 19, 44, 61, 67, 85, 91, 97, 107, 109, 126, 135, 137, 141, 183) were selected for further characterization.

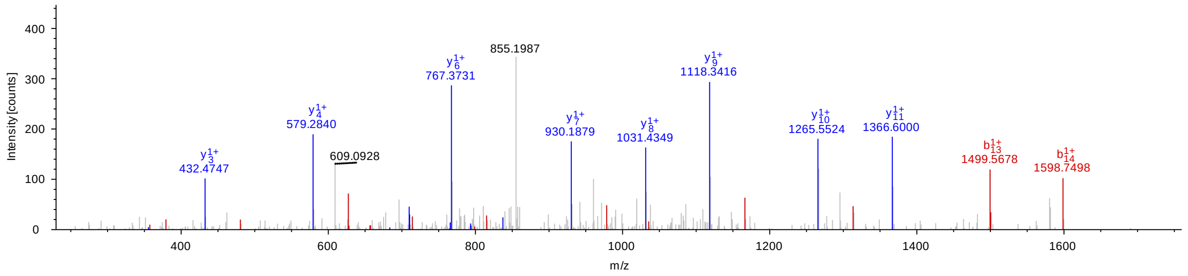

Fig. S5. A MS/MS spectrum of the digested product of scFv#m1-10 that indicates the presence of W at the V_H36-position therein. This spectrum is assigned to pentadeca peptide ASGYTFSTYGMFWVK (corresponding to the V_H24–38 amino acids): the b-ion (reflecting ASGYFSTYGMFW; *m/z* 1499.5678) and y-ion (reflecting WVK; *m/z* 432.4747) fragments are observed.

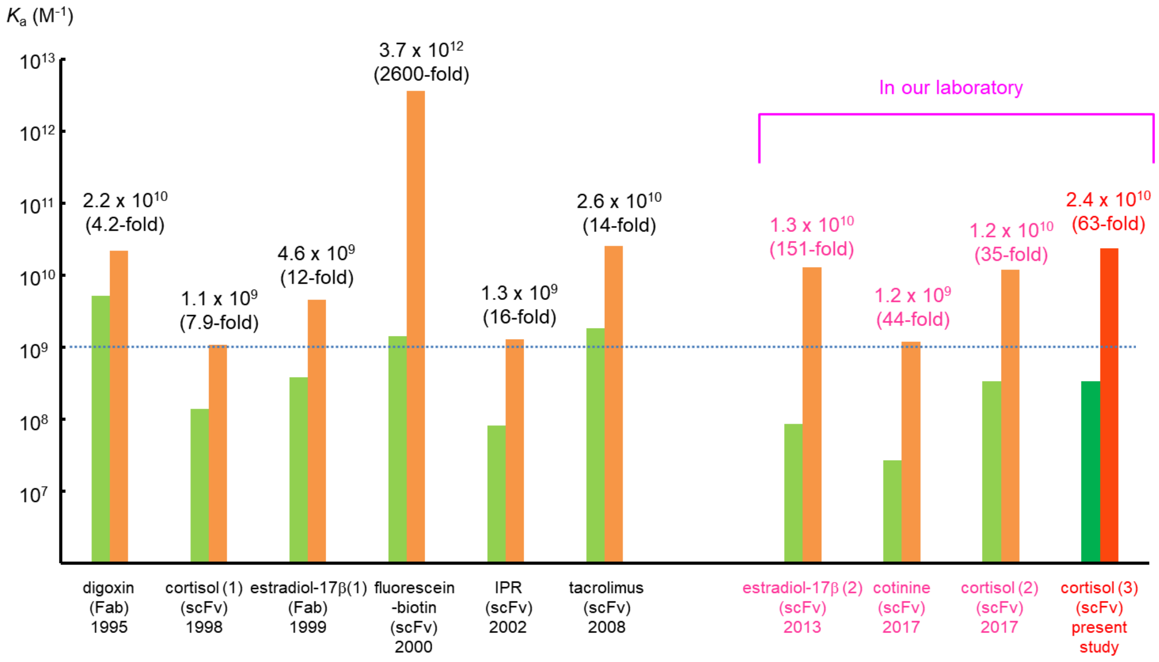

Fig. S6. Summary of selected data from affinity-maturation studies for antibody fragments targeting haptens. Data from key studies describing affinity maturation for antibody fragments (scFv or Fab) targeting haptens (shown on the abscissa) that generated a mutant with a K_a value $>10^9$ M^{-1}, with which reactive to free (not immobilized) hapten molecules, with ≥4-fold improvement, are summarized. The left (light green/green) and right (orange/red) bars show the K_a values of wild-type (prototype) antibody fragments and affinity-matured mutants, respectively. The magnitude of improvement is shown in parentheses. The associated references are as follows: digoxin[11], cortisol(1)[12], estradiol-17β(1)[13], fluorescein–biotin[14], IPR (*S*-triazine derivative)[15], tacrolimus[16], estradiol-17β(2)[8,9], cotinine[17], cortisol(2)[1], and cortisol(3) (present study). Information for some of these studies is also described in detail in our previous paper[9,18].

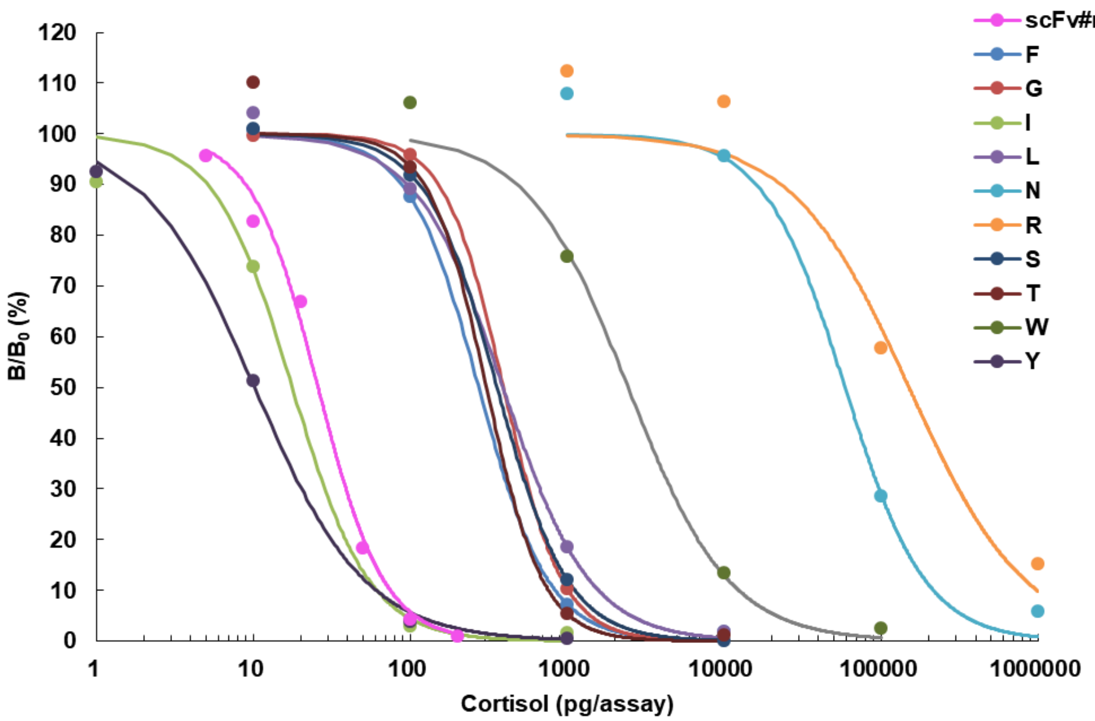

Fig. S7. Dose–response curves of ELISA using scFv#m2-97 or its mutants generated by replacing V_L49 amino acids from the original cysteine (C) in scFv#m2-97 to phenylalanine (F), glycine (G), isoleucine (I), leucine (L), asparagine (N), arginine (R), serine (S), threonine (T), tryptophan (W), and tyrosine (Y).

Table

Table S1. Cross-reactivity (%)* of the present anti-cortisol scFvs in ELISA

Steroid**	scFv					
	wt	#m1-10	#m1-18	#m2-4	#m2-91	#m2-97
Cortisol	100	100	100	100	100	100
Corticosterone	0.17	1.4	0.92	4.8	2.3	2.1
Cortisone	45	16	16	34	18	18
11-Deoxycortisol	160	110	120	220	140	140
17α-Hydroxyprogesterone	2.1	8.2	9.2	27	11	14
21-Hydroxyprogesterone	0.17	3.2	3.5	10	5.4	4.0
Progesterone	<0.01	<0.05	<0.05	0.14	0.06	<0.05
Aldosterone	<0.01	<0.01	<0.01	<0.01	<0.01	<0.01
Testosterone	<0.01	<0.001	<0.001	<0.001	<0.001	<0.001

* Calculated by the 50% displacement method[19].

** Chemical structures of which are shown in Fig. S1.

The five kinds of affinity-matured mutants referred above showed somewhat inferior recognition around the 18- and 21-hydroxy groups (as shown with corticosterone, 17α-hydroxyprogesterone, and 21-hydroxyprogesterone) compared to wt-scFv, while recognition around the 11-position was improved (as shown with cortisone and 11-deoxycortisol).

References

1. Oyama, H. *et al*. A single-step "breeding" generated a diagnostic anti-cortisol antibody fragment with over 30-fold enhanced affinity. *Biol. Pharm. Bull.* **40**, 2191–2198 (2017).
2. Kiguchi, Y. *et al*. Antibodies and engineered antibody fragments against M13 filamentous phage to facilitate phage-display-based molecular breeding. *Biol. Pharm. Bull.* **41**, 1062–1070 (2018).
3. Skerra, A. & Plückthun, A. Assembly of a functional immunoglobulin Fv fragment in *Escherichia coli*. *Science* **240**, 1038–1041 (1988).
4. Bird, R. E. *et al*. Single-chain antigen-binding proteins. *Science* **242**, 423–426 (1988).
5. Oyama, H. *et al*. *Gaussia* luciferase as a genetic fusion partner with antibody fragments for sensitive immunoassay monitoring of clinical biomarkers. *Anal. Chem.* **87**, 12387–12395 (2015).
6. Whitlow, M. *et al*. An improved linker for single-chain Fv with reduced aggregation and enhanced proteolytic stability. *Protein Eng. Des. Sel.* **6**, 989–995 (1993).
7. Oyama, H. *et al*. Anti-idiotype scFv–enzyme fusion proteins: A clonable analyte-mimicking probe for standardized immunoassays targeting small biomarkers. *Anal. Chem.* **85**, 11553–11559 (2013).
8. Kobayashi, N. *et al*. Two-step in vitro antibody affinity maturation enables estradiol-17β assays with more than 10-fold higher sensitivity. *Anal. Chem.* **82**, 1027–1038 (2010).
9. Oyama, H., Yamaguchi, S., Nakata, S., Niwa, T. & Kobayashi, N. "Breeding" diagnostic antibodies for higher assay performance: A 250-fold affinity-matured antibody mutant targeting a small biomarker. *Anal. Chem.* **85**, 4930–4937 (2013).
10. Scatchard, G. The attractions of proteins for small molecules and ions. *Ann. N. Y. Acad. Sci.* **51**, 660–672 (1949).
11. Short, M. K., Jeffrey, P. D., Kwong, R. F. & Margolies, M. N. Contribution of antibody heavy chain CDR1 to digoxin binding analyzed by random mutagenesis of phage-displayed Fab 26-10. *J. Biol. Chem.* **270**, 28541–28550 (1995).
12. Chames, P., Coulon, S. & Baty, D. Improving the affinity and the fine specificity of an anti-cortisol antibody by parsimonious mutagenesis and phage display. *J. Immunol.* **161**, 5421–5429 (1998).

13. Lamminmäki, U. *et al*. Expanding the conformational diversity by random insertions to CDRH2 results in improved anti-estradiol antibodies. *J. Mol. Biol.* **291**, 589–602 (1999).
14. Boder, E. T., Midelfort, K. S. & Wittrup, K. D. Directed evolution of antibody fragments with monovalent femtomolar antigen-binding affinity. *Proc. Natl. Acad. Sci. U. S. A.* **97**, 10701–10705 (2000).
15. Kramer, K. Evolutionary affinity and selectivity optimization of a pesticide-selective antibody utilizing a hapten-selective immunoglobulin repertoire. *Environ. Sci. Technol.* **36**, 4892–4898 (2002).
16. Siegel, R. W., Baugher, W., Rahn, T., Drengler, S. & Tyner, J. Affinity maturation of tacrolimus antibody for improved immunoassay performance. *Clin. Chem.* **54**, 1008–1017 (2008).
17. Oyama, H. *et al*. One-shot in vitro evolution generated an antibody fragment for testing urinary cotinine with more than 40-fold enhanced affinity. *Anal. Chem.* **89**, 988–995 (2017).
18. Kobayashi, N. & Oyama, H. Antibody engineering toward high-sensitivity high-throughput immunosensing of small molecules. *Analyst* **136**, 642–651 (2011).
19. Abraham, G. E. Solid-phase radioimmunoassay of estradiol-17β. *J. Clin. Endocrinol. Metab.* **29**, 866–870 (1969).
